# The Emboless® venous chamber efficiently reduces air bubbles: a randomized study of chronic hemodialysis patients

**DOI:** 10.1093/ckj/sfae323

**Published:** 2024-10-24

**Authors:** Ulf Forsberg, Per Jonsson, Bernd Stegmayr

**Affiliations:** Department of Public Health and Clinical Medicine, the Skelleftea Unit, Umea University, Umea, Sweden; Department of Public Health and Clinical Medicine, Unit of Medicine, Umea University, Umea, Sweden; Department of Public Health and Clinical Medicine, Unit of Medicine, Umea University, Umea, Sweden

**Keywords:** bio-compatibility, cardiovascular, hemodiafiltration, hemodialysis, thrombosis

## Abstract

**Background:**

When blood passes the extracorporeal circuit, air microbubbles (MBs) contaminate the blood. Some MBs will end up as microemboli in the lung, heart, and brain. MB exposure has no medical purpose and is considered to be bio-incompatible. Selecting venous chambers with a high removal rate of MBs is warranted to reduce the risks of air bio-incompatibility. The primary aim was to compare the Fresenius 5008 (F5008-VC) and the Emboless^®^ (Emboless-VC) venous chambers regarding the elimination of MBs in the return bloodline during hemodialysis (HD).

**Methods:**

Twenty patients underwent 80 sessions of cross-over HD using both the F5008-VC and the Emboless-VC randomized such that half started with the F5008-VC and half with the Emboless-VC. For 32 of the 80 sessions, measurements were also performed during hemodiafiltrations (HDF) after the initial HD. MBs were measured with an ultrasound device (within the size range of 20–500 µm) at the “inlet” and “outlet” bloodline of the venous chambers. The Wilcoxon pairwise test compared the percentage of MB elimination between venous chambers.

**Results:**

During HD, the median reduction of MBs for the outlet was 39% with the F5008-VC and 76% with the Emboless-VC (*P *< .001). During HDF, the reduction was 28% with the F5008-VC and 70% with the Emboless-VC (*P *< .001).

**Conclusion:**

Fewer MBs and subsequently fewer microemboli entered the bloodline of the patient using the Emboless-VC compared to the F5008-VC venous chamber during HD and during HDF. Venous chambers with a high removal rate of MBs will reduce the extent of air emboli.

## Graphical Abstract

**Graphical Abstract:**
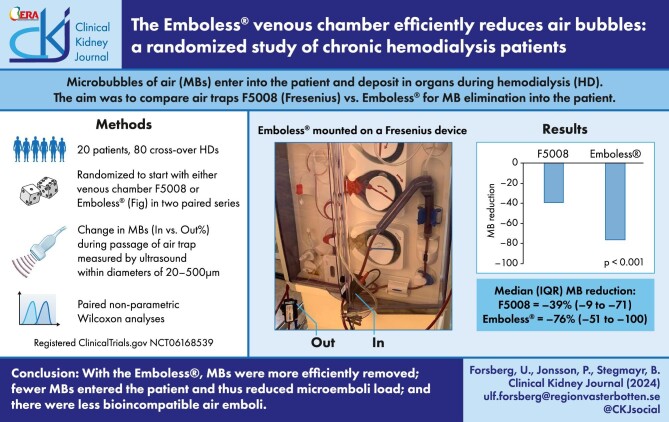

KEY LEARNING POINTS **What was known:**

- Microbubbles of air contaminate the hemodialysis extracorporeal bloodline.
- Microbubbles frequently pass the venous chamber and enter the patient without inducing a safety alarm.
- As such, microemboli deposit in organs such as the lung, heart, and brain.

**This study adds:**

- The clinical study investigated the extent of microbubble (20–500 µm diameter) exposure into the return bloodline of the patient.
- Venous chambers have different removal efficacy.
- The Emboless® venous chamber (Emboless-VC) removed 76% of microbubbles whereas the Fresenius 5008 venous chamber (F5008-VC) removed 39% (*P *< .001).

**Potential impact:**

- Patients are exposed to numerous air microbubbles.
- Requests for efficient venous chambers are crucial to limit the exposure of microbubbles altering bio-compatibility and ending up as air emboli causing organ damage.
- In this study, the Emboless-VC reduced microbubbles to a greater extent and subsequent air emboli during dialysis compared to the F5008-VC.

## INTRODUCTION

Hemodialysis (HD) patients have an increased morbidity and shortened life expectancy [1, 2]. During HD, the blood of the patient passes the extracorporeal circuit (ECC) that contains a venous chamber to prevent air from passing through the return bloodline into the patient.

Venous chambers (VC) in clinical use have limited capacity in preventing air microbubbles (MBs) from entering the return bloodline [2–6]. MBs that enter the blood vessel activate the coagulation cascade [7]. MBs were verified as microemboli at autopsy within the lung, heart, and brain tissues of HD patients [8, 9]. Such microemboli correlated with prolonged HD vintage time [9]. Thus, the ability to prevent MB leakage from the ECC into the blood vessels of HD patients is inadequate, and the risk exposure is as frequent and prolonged as a respective patient's treatment.

Leakage of air into the blood of patients during HD is a risk commonly addressed in the standards for dialysis machines (IEC60601-2-16) [10]. However, state of the art technology is unable to manage this risk by inherent design. The Standard states that “the haemodialysis equipment shall include a protective system to protect the patient from air infusion, under normal condition and single fault condition, that may cause a hazardous situation.” The Standard also states that “there was not enough scientific literature to define a safe alarm limit in this particular Standard.” “Exposure to MBs should be taken into account and possible preventive measures should be considered by the manufacturers’ risk management process” [10]. Ultimately, each manufacturer must justify an alarm limit that prevents the acute effects of air infusion.

*In vitro* data exist regarding the capacity of VC for reducing MB content [11–13]. A recent *in vitro* study showed a limited capacity of MB removal for four VC in clinical use (for devices Fresenius 5008, Fresenius 6008, Baxter AK98, and Artis); the best of these was the F5008-VC [14]. That study also included a preclinical venous chamber Emboless^®^ (Emboless-VC) that showed the best overall reduction of MBs from the return bloodline [14].

**Figure 1:**
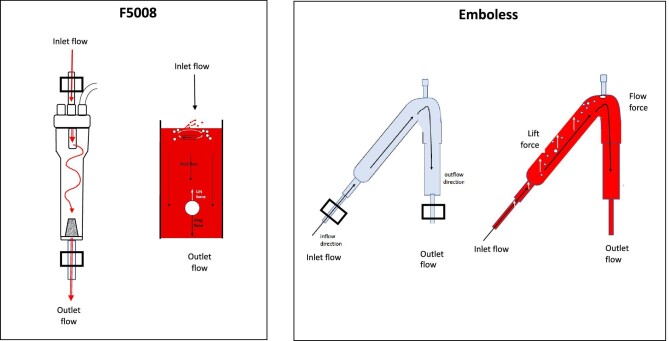
The VCs F5008-VC (left panel) and Emboless-VC (right panel) displaying the bubble probe locations (open square) at the inlet and outlet bloodlines as well as the flow directions. To the right of each is a schematic drawing showing the air bubble development and directions with a whipped cream effect, as well as lift forces (white arrow) versus drag forces (black arrow), and fluid flow forces (black arrow).

The primary aim was to compare the F5008-VC with the Emboless-VC (Fig. 1) regarding the capacity to reduce MBs in the return bloodline during HD. Aside from safety issues, a secondary aim was to clarify whether the outcome differs during hemodiafiltration (HDF).

## MATERIALS AND METHODS

### Study design

**Figure 2:**
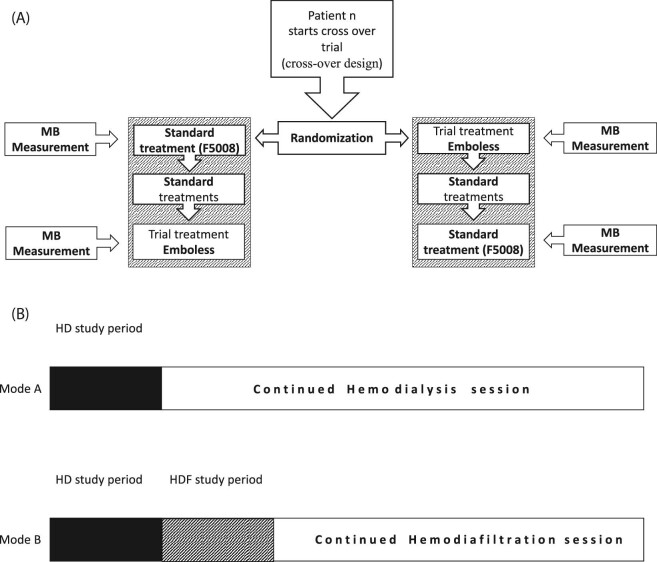
(**A**) Randomization cross-over design and MB measurements. (**B**) Mode A shows a patient undergoing only HD. The study dialysis shows that microbubble measurement was performed during the first 30 minutes (blackened area), and thereafter, the patient continued his/her HD without measurement (white area). Mode B shows a patient on regular HDF. For this mode measurement, of bubble content was performed for HD (blackened area) during the first 30 min. Then the procedure was switched to HDF (hatched area) and measurement of bubble content performed for another 30 min. Thereafter the patient continued his/her regular HDF without measurement (whitened area).

The study was a cross-over HD design using two types of venous blood line, of which patients used both types in a randomization of the type to start treatment with. Thus, the patient was his/her own control (Fig. 2A and B).

### Blood line and dialysis setting

One type of blood line used was the LifeLine beta, AV-Set ONLINEplus BVM 5008-R (F5008-VC) by Fresenius Medical Care, Bad Homburg, Germany. The second type of blood line was the Emboless^®^ (Emboless-VC) by Embody AB, Umea, Sweden.

When using the Emboless-VC, extra space is required. This causes the front door of the device to be slightly ajar. A stoppage of the blood pump is prevented by two adapters for function, safety, and user interface. The Emboless-VC bloodline concept was accepted as an inhouse product under the former Swedish regulation (SOSFS2008:1) [15] by the healthcare institution Västerbotten County Council, Sweden. The production of the Emboless-VC bloodline for clinical test was by a third-party manufacturer that holds a quality system according to ISO 13485 [16] and has a CE-marked bloodline for the EU market. The arrangement was accepted by the Swedish Medical Product Agency along with the study plan.

Fifteen dialysis machines were used that were all the 5008 Cordiax brand (Fresenius Medical Care, Bad Homburg, Germany). Dialyzers were provided by Fresenius Medical Care (Supplement Table 1).

The intent was to use a blood pump speed (Qb) of 300 ml/min. In series with limited access flow, the Qb had to be reduced usually to the same level within the pairs. The Qb was set at a mean of 297 (±8.1, median 300, range 260–300 ml/min).

### Study population

Twenty chronic HD patients were included in a cross-over randomized study to compare the efficacy of microbubble removal of the Emboless-VC versus the F5008-VC (Fig. 1) during HD.

Each patient underwent a total of four dialyses divided into two separate pairs (Fig. 2A). For each pair, a randomization decided which of the VC was the first choice. Accordingly, 20 consecutive patients underwent 80 study dialyses.

The inclusion criteria were any patient (irrespective of gender or ethnicity) undergoing chronic dialysis on site due to end stage renal disease of any reason and as follows: 18 years and older and accepted by consent to participate in the study. The exclusion criteria were patients not expected to fulfill a whole series of two pairs of dialyses within the study, e.g. active cancer, severe infection, cachexia, or planned-for kidney transplantation.

Dialyses were administered unblinded for the staff and the patients regarding the type of venous chamber used. MB data were stored in the computer until downloaded. Statistical analyses were blinded.

The study was approved by the Ethical committee, Umea (Dnr 09–097 M, 3 July 2009), the Ethical Authority, Sweden (20 February 2020), the Medical Product Agency, Sweden (Dnr 5.1–2021-42949, CIV-20-02-031667), and ClinicalTrials.gov (NCT06168539) in accordance with the Declaration of Helsinki [17]; patients were informed and signed consents.

### Data collection—microbubbles (MB)

During HD, MBs were counted using an ultrasound device (BCC 200) that included two channels and two probes made for 1/5-inch bloodlines and calibrated for the type of bloodlines used in the present study (GAMPT mbH, Merseburg, Germany). One probe for data collection of MBs was set at the inlet bloodline (inlet) to the venous chamber and the other at the outlet bloodline (outlet, Fig. 1). The outlet represented data of MBs in the blood that returned to the patient.

The BCC 200 measures the numbers of microbubbles and their diameter size within the range of 20 to 500 µm and as “overrange” >500 µm.

Measurements of MBs were performed during HD for at least 30 min, or up to 90 min, with the intent to achieve a total number of MBs of >1000 inlet counts. All patients underwent study HD. If the standard treatment of the patient was HDF, the device mode after HD was shifted to HDF for another 30 min (Fig. 2B).

Data used for analyses were:

1) Size groups (*n* = 97 data points/dialysis): data recording started with a single size of 20 µm (representing all up to 20 µm), and thereafter in increments of 5 µm that represented one size group, e.g. 21–25 µm, 26–30 µm, and up to 496–500 µm.
2) Three merged subgroups were formed by size groups: “small” (20–199 μm diameter, *n* = 36 data points/dialysis), “medium” (200–299 μm, *n* = 20 data points/dialysis), and “large” (300–500 μm, *n* = 41 data points/dialysis).
3) ‘Overrange’ data, representing all MBs with a diameter >500 µm (*n* = 1 data point/dialysis).
4) The total number (*n* = 1 data point/dialysis): includes values from all size groups and the overrange.

Analysis revealed the numbers of MB counts at the inlet (MB_Inlet_) and at the outlet (MB_Outlet_). The efficacy to eliminate MBs was calculated by the change in MBs as a percentage (ΔMB%) between the outlet minus the inlet to the venous chamber according to the formulas (1)–(3):


(1) \begin{equation*}{\mathrm{\Delta MB\% = 100 \times }}\left( {{\mathrm{M}}{{{\mathrm{B}}}_{{\mathrm{Outlet\ }}}}{\mathrm{ - M}}{{{\mathrm{B}}}_{{\mathrm{Inlet\ }}}}} \right){\mathrm{/M}}{{{\mathrm{B}}}_{{\mathrm{Inlet\ }}}}\end{equation*}

A reduction of MBs would be:

Given MB_Inlet_ = 20 and MB_Outlet_ = 10, Equation (1) results in


(2) \begin{equation*}{\mathrm{\Delta MB\% = 100 \times (10 - 20)/20 = > \Delta MB\% = - 50\% ,}}\end{equation*}

Given MB_Inlet_ = 20 and MB_Outlet_ = 40, Equation (1) results in


(3) \begin{equation*}{\mathrm{\Delta MB\% = 100 \times (40 - 20)/20 = > \Delta MB\% = + 100\% ,\ }}\end{equation*}

To allow mathematical calculation, when lacking any inlet count but having Outlet MB counts, an imputation was made to achieve a change in MBs close to +100% increase (i.e. figures used to show an imputation for inlet of 0.5 for an outlet of 1, 0.9 for 2, 1.5 for 3 and 1.9 for ≥4). Imputations for HD data (maximal data points are *n* = 3960) the F5008-VC were 28 (7‰) and for Emboless-VC 27 (7‰), and for HDF (maximal data points *n* = 1584) the F5008-VC were 15 (9‰) and for Emboless-VC 1 (1‰). If no count was present in either the inlet or the outlet, no difference was calculated and the group was omitted from analysis as missing value [for HD and F5008-VC *n* = 73 (2%) and Emboless-VC *n* = 77 (2%), and for HDF for F5008-VC 3 (2‰) and for Emboless-VC *n* = 35 (2%)]. A maximum of 100% MBs could be removed, while at the Outlet MBs could exceed 100%.

### Safety issues including further laboratory follow-up (see also the Supplement).

Blood was drawn pre-dialysis at 30 min, and at 180 min during dialysis for analyses of e.g. erythrocytes, leukocytes, and platelets. Safety analysis included dialysis protocols, technical measures, blood pressure, experienced side effects, visual grading of clotting of the dialyzer and the venous chamber, recovery time after HD according to Rayner *et al.* [18], and heparin doses/kg bow.

Prior to, during, and after the study, monitoring of the safety routines and study compliance was performed by independent monitoring through the Clinical Research Center at the hospital.

### Statistical analyses

Based on paired non-parametric statistics, a sample size of 60 paired dialyses were estimated to give a power of 90% and an alfa of .05 to detect a different efficacy of microbubble removal by the VC. Intermediate safety analyses were planned.

The use of all 20 patients as their own controls was estimated to represent significant variation between subjects. This motivated us to use the same patients for another series, since the deviations between series with patients being their own controls give less confounding results than using one person as the case and another as the control, even adjusting for age, sex, and eventually one more variable.

Comparisons were performed between the change in percentage of outlet versus inlet counts adjusted for a data collection time of 30 min. Data were analyzed separately for HD and HDF and respectively by size groups. The medians and inter-quartile ranges (IQR) are given for non-parametric analyses and the means and standard deviations (±) for parametric analyses.

Comparisons were done between the two different VC using non-parametric Wilcoxon paired analyses and the Mann–Whitney test for non-paired data due to the possibility of skewed distributions. A two-tailed value of *P *< .01 was defined as significant for the primary end point to cease the study. A *P* value <.05 was considered significant for single comparisons. For stepwise multiple regression analysis including ANOVA analysis, variables included in the model had a *P *< .015 for the univariate correlation by the Spearman test or by group analyses. In addition, age and sex were included in the analyses irrespective of a lack of a significant difference.

## RESULTS

The safety committee performed comparative analysis between the F5008-VC and the Emboless-VC for the exposure of MBs to the patient. Safety evaluation exhibited significantly better MB elimination with the Emboless-VC than for the F5008-VC (*P *< .001), which resulted in ending the study after 80 participants included HD (initially 120 dialyses were intended).

### Patient characteristics

Twenty patients (mean age 71 ± 12 years, 60% men) that underwent 80 dialyses were included. The diagnoses leading to end stage kidney disease were glomerulonephritis (*n* = 4), diabetes mellitus (*n* = 3), polycystic kidney disease (*n* = 3), nephrosclerosis (*n* = 3), and one patient each for congenital urethra valvula with hydronephrosis, AL-amyloidosis, myeloma, light chain disease, chronic pyelonephritis, nephrectomy due to renal cancer, and lithium nephropathy. For information on vascular access, dialysis devices, dialyzers, dialysates, anticoagulation, and blood pump speed see the Supplement.

### Hemodialysis: primary outcome

**Figure 3:**
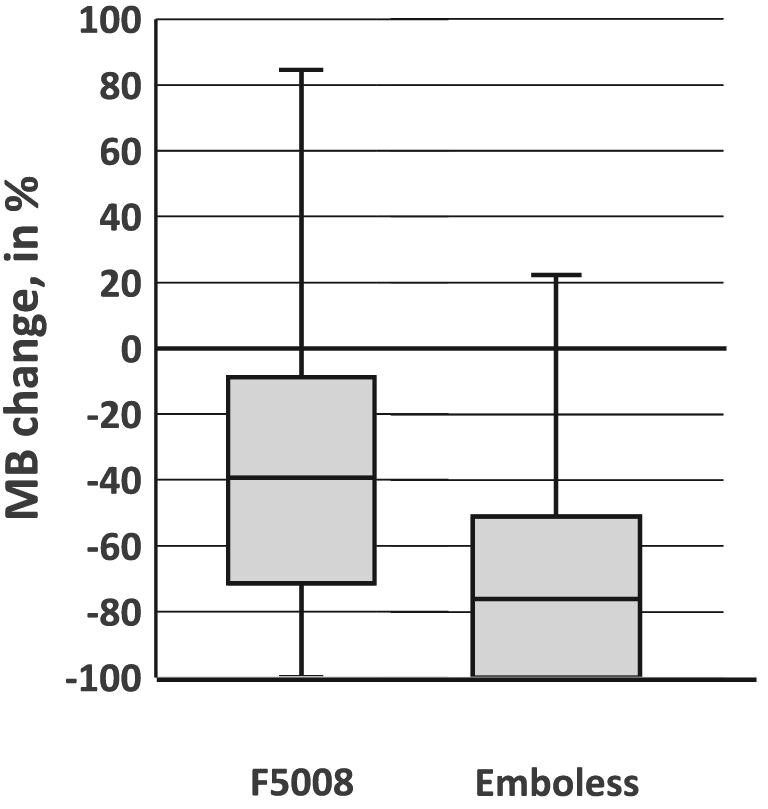
Box plot of the median and quartiles of change in MBs during HD at the outlet versus the inlet (as a percentage) for the Emboless-VC and F5008-VC. Negative values on the *y*-axis indicate a reduction (elimination) of MBs and positive values indicate an increase in MBs.

**Figure 4:**
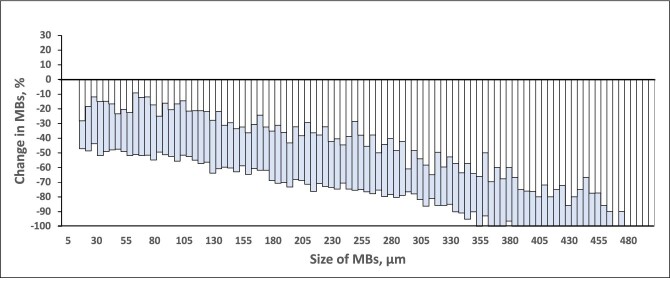
Median change in MBs according to size at the outlet versus the inlet (in percentage) during HD. Negative values on the *y*-axis indicate a reduction (elimination) of MBs and positive values indicate an increase in MBs. Each staple in the figure represents the value for the size group of 5 µm. The white staples represent the F5008-VC values overlaid on the gray (blue) staples that represent the Emboless-VC values.

For patients during HD, the median change (ΔMB%) of MBs in the Outlet (MB_Outlet_) versus the Inlet (MB_Inlet_) was −39% with the F5008-VC (size group data points *n* = 3807) and −76% with the Emboless-VC (*n* = 3805), resulting in 3740 pairs for comparison (*P *< .001, Table 1, Fig. 3). Figure 4 shows the distribution of all MB size groups between 20 and 500 µm.

**Table 1:** HD: comparison of 40 pairs of data for the 40 series using the F5008-VC versus the 40 series of Emboless-VC, in a total of 80 dialyses for all patients.

	Pairs	HD-F5008-VC	HD-Emboless-VC	*P* value
MBs inlet/30min	40	2318 (1216 to 4887)	2151 (1418 to 4303)	.657
MBs outlet/30min	40	1445 (834 to 3516)	962 (409 to 1386)	.007
**ΔMB_Outlet-Inlet_, %:**				
Total ΔMB_Outlet-Inlet_, %	40	−33 (−24 to −40)	−62 (−52 to −74)	<.001
Overrange ΔMB_Outlet-Inlet_, %	40	−100 (−100 to −100)	−100 (−100 to −100)	.003^a^
**Size groups of 5 µm:**				
**All** ΔMB_Outlet-Inlet_, %	3740	−39 (−9 to −71)	−76 (−51 to −100)	<.001
**Small** ΔMB_Outlet-Inlet_, %	1439	−24 (0 to −41)	−56 (−39 to −71)	<.001
**Medium** ΔMB_Outlet-Inlet_, %	800	−40 (−15 to −64)	−75 (−58 to −89)	<.001
**Large** ΔMB_Outlet-Inlet_, %	1501	−70 (−28 to −100)	−100 (−83 to −100)	<.001

a Best elimination by the Emboless-VC (F5008-VC: displayed as mean −78% ± 123% versus Emboless-VC: 98% ± 14%).

The exposure of MBs is given as the median (IQR) numbers at the inlet and outlet and adjusted to the HD time in30 minutes. ΔMB_Outlet-Inlet_ represents the median change (IQR) as a percentage of MBs from the inlet to the outlet of the F5008-VC and Emboless-VC. Data are presented for the 40 pairs as the total and for the overrange (for sizes >500 µm). Data are also expressed for size groups of 5 µm diameter in the range of 20–500 µm diameter for All (the whole span) and differentiated for small (20–199 µm), medium (200–299 µm), and large (300–500 µm). The median change of ΔMB_Outlet-Inlet_ is given as a percentage (a negative value represents a reduction of MBs at the outlet). A −100% change represents a total elimination of MBs.

### Hemodialysis: subgroup analysis

For the F5008-VC versus the Emboless-VC, the median reductions of counts at the outlet bloodline for the various MB sizes were as follows: small −24% versus −56% (*P *< .001), medium −40 versus −75% (*P *< .001), and large −70 versus −100% (*P *< .001, Table 1).

The number of MBs in 30 min at the inlet did not differ between the VC (Table 1).

### Hemodialysis: stepwise multiple regression analysis

MB reduction during HD was related to the variables VCs F5008-VC versus Emboless-VC (*r* = 0.648, *P *< .001, Supplement Table 2). Excluded by the analysis were the variables sex, age, hematocrit, dose of anticoagulation/kg body weight, inlet bubbles in 30 min, and platelet and leukocyte count at the start. Kept in the final calculation were sex, age, inlet bubbles in 30 min, and venous chamber type.

The inlet MB number in 30 min during HD was inversely related to the arterial pressure measured between the access and the blood pump (*r* = 0.336, *P *= .004, Supplemental Table 3). Excluded by the analysis were the following variables: platelet count at the start and at 180 min, leukocytes at the start, as well as the non-significant variables in the univariate analysis, i.e. the F5008-VC versus Emboless-VC, sex, and age.

### Hemodiafiltration (HDF): secondary outcome

**Figure 5:**
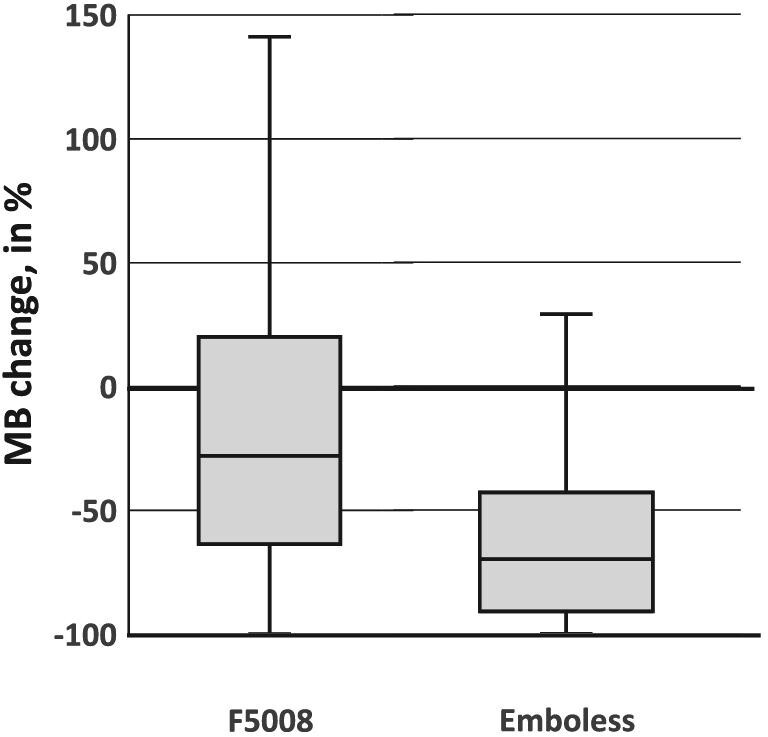
Box plot of the median and quartiles of change in MBs during HDF at the outlet versus the inlet (in percentage) for the Emboless-VC and F5008-VC. Negative values on the *y*-axis indicate a reduction (elimination) of MBs and positive values indicate an increase in MBs.

**Figure 6:**
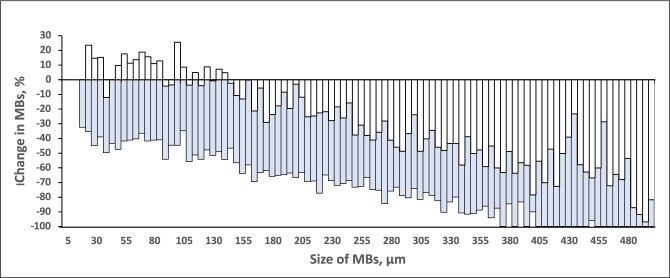
Median change in MBs according to size at the outlet versus the inlet (in percentage) during HDF. Negative values on the *y*-axis indicate a reduction (elimination) of MBs and positive values indicate an increase in MBs. Each staple in the figure represents the value for the size group of 5 µm. The white staples represent the F5008-VC values overlaid on the gray (blue) staples that represent the Emboless-VC values.

For patients during HDF, the median ΔMB% was −28% with the F5008-VC (*n* = 1549) and −70% with the Emboless-VC (*n* = 1517) resulting in 1514 pairs for statistical comparison (*P *< .001). The ΔMB% values during HDF are shown in Figs 5 and 6.

### Hemodiafiltration: subgroup analysis

For the F5008-VC versus the Emboless-VC, the median ΔMB%s for the various MB sizes were as follows: small ±0% versus −51% (*P *< .001), medium −29 versus −72% (*P *< .001), and large −57 versus −94% (*P *< .001, Table 2).

**Table 2:** HDF: comparison of 16 pairs of data for the F5008-VC versus the Emboless-VC for all patients.

	Pairs	HDF- F5008-VC	HDF-Emboless-VC	*P* value
MBs inlet/30min	16	2991 (1800 to 5246)	3833 (1466 to 6950)	.255
MBs outlet/30 min	16	2859 (1478 to 4550)	1753 (722 to 3162)	.163
**ΔMB_Outlet-Inlet_** %				
Total ΔMB_Outlet-Inlet_, %	16	−12 (16 to −36)	−61 (−47 to −71)	.002
Overrange ΔMB_Outlet-Inlet_, %	16	−99 (−99 to −100)	−100 (−100 to −100)	.12
**Size groups of 5 µm:**				
**All** ΔMB_Outlet-Inlet_, %	1514	−28 (20 to −64)	−70 (−43 to −91)	<.001
**Small** ΔMB_Outlet-Inlet_, %	576	±0 (42 to −33)	−51 (−28 to −66)	<.001
**Medium** ΔMB_Outlet-Inlet_, %	319	−29 (14 to −64)	−72 (−57 to −82)	<.001
**Large** ΔMB_Outlet-Inlet_, %	649	−57 (0 to −91)	−94 (−70 to −100)	<.001

The exposure of MBs is given as the median (IQR) numbers at the inlet and outlet and adjusted to the HDF time in 30 min. ΔMB_Outlet-Inlet_ represent the median change (IQR) in percentage of MBs from the inlet to the outlet of the F5008-VC and Emboless-VC. Data are presented for the 16 pairs as the total and for overrange (for sizes >500 µm). Data are also expressed for size groups of 5 µm diameter in the range of 20–500 µm diameter for all (the whole span) and differentiated for small (20–199 µm), medium (200–299 µm), and large (300–500 µm). The median change of ΔMB_Outlet-Inlet_ is given as a percentage (a negative value represents a reduction of MBs at the outlet). A −100% change represents a total elimination of MBs.

### HD versus HDF

When comparing results for HD versus HDF, for the Emboless-VC there was no significant difference in the ΔMB%, whereas for the F5008-VC there was a significantly lower elimination of MBs for the HDF compared to the HD sessions (Table 1 versus Table 2).

### Safety outcome and other variables (see also the Supplement)

No side effects were recorded regarding the interaction between the dialysis machine and the Emboless-VC bloodline. Further, no air alarm was activated during any of the 80 dialyses. The Supplement contains information about the leukocyte and platelet concentrations adjusted for the change in hematocrit by ultrafiltration according to Schneditz *et al.* [19], heparin doses, bloodline clotting, and recovery time.

## DISCUSSION

The primary aim of the present study was to evaluate whether the extent of MBs that appear in the extracorporeal circuit at the outlet line during HD changed differently for the F5008-VC versus the Emboless-VC. Overall, the Emboless-VC reduced MBs more efficiently than the F5008-VC; this was valid for the small, medium, and large-sized MBs. During HDF, MBs were more effectively reduced by the Emboless-VC compared to the F5008-VC. To our knowledge, this is the first clinical comparative study that gives information of MB reduction capacity of VC. Our data are similar to the results of an *in vitro* study that compared the aforementioned VC [14]. A net increase in MBs at the outlet versus the inlet may be due to (i) splitting of large MBs into several smaller ones, (ii) the addition of bubbles from air within the chamber, or both (i) and (ii).

Comparison between the two systems showed few dialyses with symptoms, and no episode differed from the routine procedures.

A higher exposure to MBs at the inlet was related to the negative pressure in the arterial line. The negative pressure may enable more air to be sucked into the bloodline at the site of the access, such as if there existed cracked or insufficiently closed connections, pressure lines, and heparin infusion lines if not primed with fluid [20] or insufficient auto priming [21]. Other factors could be that MBs are caused by cavitations induced by a high negative pressure [4] or retained air from the dialyzer [21].

In the present study, reductions of leukocytes and platelets were noted during dialysis with both bloodline systems. This may well be based on the similar dialyzer blood–membrane interaction and air contamination prior to the VC. Air contamination prior to the venous chamber has been shown previously [6, 21]. Also, activation of clotting has been known to appear within the dialyzer. However, insufficient MB elimination by the VC cause additions of air bubbles that enter the bloodline of the patients. Those MBs will end up as microemboli and present a thrombotic burden to the patient. The extent of subclinical thromboses in the course of HD is only partly resolved by fibrinolysis throughout the days between the dialyses [22], and residuals will contribute to tissue damage.

The exposure of MBs via entry into the veins of the patient occurs during the whole dialysis [13, 23]. The autopsy studies confirm activated clotting and MBs deposited as microemboli [8, 9, 24] that are also related to the vintage time on HD [9]. Such additive damage may well support the shortened life expectancy and pathophysiologic specific findings of tissue damage as mentioned by others such as pulmonary fibrosis, pulmonary arterial hypertension, increased prevalence of cerebral stroke, cerebral atrophy, and silent myocardial infarctions and myocardial stunning; such findings are less frequent in peritoneal dialysis patients [9].

According to the Standard for risk management to medical devices (ISO14971) [25], the management should take into account available information of new technical achievements. The MB exposure has no medical purpose and can be defined as bio-incompatibility. According to the risk assessment in the Standard for biological evaluation of medical devices (ISO 10993) [26], multiple repeated exposures of useless air bubbles with subsequent coagulation and microemboli have to be taken into account [9]. The “risk exposure” mentioned in the ISO 10993 [26] is as frequent and prolonged as the time a respective patient undergoes HD.

An example of foreseeable misuse is the accidental opening or insufficient closing of a connection that results in increased exposure of MBs. Cases of air alarms indicate extensive increased exposure into the bloodline, and according to the low reduction in the VC in clinical use also into the patient. Therefore, a robust and effective barrier against MBs would be desirable.

The new Emboless-VC technology was not able to remove all MBs. However, the Emboless-VC exhibited a more favorable reduction of MBs than the F5008-VC. A better venous chamber would enable a lowering of the alarm limit for MBs. Thus, the combination of the Emboless-VC and a lowered limit for the micro air alarms would constitute a risk reduction “as low as reasonably possibly achievable” as suggested in the Standard ISO14971 [25]. This would enable a new state of the art.

### Limitations

The use of a paired study with the patient as his/her own control minimizes the interdialytic variables, and allows a lower number of patients while maintaining a high power of the study. Also, this minimizes the influence of external confounding variables. Still a small variation in the blood pump speed could differ by ±20 ml/min since the limited access flow did not always allow nurses to set the flow at 300 ml/min, which was the goal. The clinical condition and the extent of ultrafiltration could differ even if the intent was to use the same weekday for dialysis for both study settings.

The study supports clinicians requesting bloodline systems that cause minimal exposure of MBs into the patient. This can be achieved, for example, with appropriate connections between bloodlines, adequate priming, and selecting bloodlines with proved efficient VC.

In conclusion, the present study emphasizes the importance of the awareness of manufacturers and health institutions to minimize the risk of the exposure to MBs entering the patient during HD. The study verifies that the Emboless-VC can be seen as a risk reducing barrier that improves the safety regarding air infusion into the patient.

## Supplementary Material

sfae323_Supplemental_File

## Data Availability

The data underlying this article will be shared on reasonable request to the corresponding author.
