# Supplementary material for: The Emboless® venous chamber efficiently reduces air bubbles: a randomized study of chronic hemodialysis patients
Source: Clin Kidney J. 2024 Oct 24;17(11):sfae323. doi: 10.1093/ckj/sfae323 (PMC11579606; doi:10.1093/ckj/sfae323)
Supplement: sfae323_Supplemental_File [file sfae323_supplemental_file.docx]

**SUPPLEMENTAL MATERIAL See separate file**

**Methods (supplement) Page 2**

**Supplement Table 1 Page 2**

**Results (Supplement) Page 3-7**

**Supplement Table 2 Page 3**

**Supplement Table 3 Page 4**

**Supplement Table 4 Page 5**

**Supplement Table 5 Page 7**

**METHODS**

The arteriovenous fistula (AVF, 6 patients, 24 HDs) and the central dialysis catheter (CDC, 14 patients 56 HDs) were the accesses used. The baseline hemoglobin was 103 ±12 g/L. The dialyzers used were provided by Fresenius Medical Care (Supplement Table 1). The anticoagulants used were fondaparinux 2.5mg/dose in two patients (eight dialysis) and tinzaparin in 18 patients (72 dialysis: median dose 3500, range 2500-8000 units/H and a median of 51 units/kg bow). The same dosages were used for all four study dialyses for a respective patient.

The identity of the dialysis device was registered for each study dialysis. The dialysates used were provided by Fresenius Medical Care as Smartbag 211.25, 211.50, 311.25 or 311.50 – the first number represents the final concentration of potassium, and the third, fourth and fifth numbers represent the calcium concentration in mmol/L. Each pair of study dialyses used the same type of concentrate.

Supplement Table 1: The various dialyzers purchased from Fresenius Medical Care that were used by the patients. The intent was to use the same dialyzer type for both types of bloodlines (Emboless-VC versus F5008-VC).

| Dialyzer | Emboless-VC | F5008-VC | Total |
| --- | --- | --- | --- |
| FX80 | 24 | 24 | 48 |
| FX600 | 2 | 2 | 4 |
| FX800 | 10 | 11 | 21 |
| FX1000 | 4 | 3 | 7 |
| Total | 40 | 40 | 80 |

Blood was drawn pre-dialysis, at 30 minutes, and at 180 minutes during dialysis for laboratory analyses of changes of blood corpuscles erythrocytes, leukocytes, lymphocytes, monocytes, eosinophils, neutrophils, platelets, and albumin. Changes were corrected for eventual effect of ultrafiltration. Each dialysis included data of technical measures, blood pressure and eventual side effects. Also included were visual grading of clotting of the dialyzer as none (grade 0), slight signs (grade 0.5), slight (grade 1), medium (grade 2), moderate (grade 3), or severe (clotting that would need restart- grade 4). After HD, grading for clots in the venous chamber was made as follows: none (grade 0), rim at surface (grade 1), visible clot at the outlet filter (grade 2), or clot with stop of flow (grade 3).

**RESULTS**

The intent was to use a blood pump speed (Qb) of 300 ml/min. In some series with limited access flow, the Qb had to be reduced usually to the same level within the pairs. The Qb was set at a mean of 297 (±8.1, median 300, range 260-300 ml/min).

Supplement Table 2: Stepwise multiple regression analysis with the extent of MB reduction in % as the dependent factor. Excluded in the final calculation were the variables sex, age, Inlet bubbles/30min.

|  | | | | | | | | | |
| --- | --- | --- | --- | --- | --- | --- | --- | --- | --- |
| Model | R | R Square | Adjusted R Square | Std. Error of the Estimate | Change Statistics | | | | |
|  |  |  |  |  | R Square Change | F Change | df1 | df2 | Sig. F Change |
| 1 | 0.648^a^ | 0.420 | 0.413 | 17.63 | 0.420 | 56.590 | 1 | 78 | <0.001 |
| a. Predictors: (Constant), venous chamber F5008-VC vs Emboless-VC | | | | | | | | | |

Supplement Table 3: Stepwise multiple regression analysis with the Inlet extent of MBs/30min during HD as the dependent factor. The remaining significant variable was the extent of the inverse arterial pressure measured between the access and the blood pump.

| Model | R | R Square | Adjusted R Square | Std. Error of the Estimate | Change Statistics | | | | |
| --- | --- | --- | --- | --- | --- | --- | --- | --- | --- |
|  |  |  |  |  | R Square Change | F Change | df1 | df2 | Sig. F Change |
| 1 | 0.336^a^ | 0.113 | 0.100 | 5147.5 | 0.113 | 9.039 | 1 | 71 | 0.004 |
| **a. Predictors: (Constant), Arterial pressure** | | | | | | | | | |

When comparing HD with HDF for the same venous chamber, including only the same patient, for the ‘Small’ MBs the reduction for the F5008-VC was better for HD versus HDF (n=576 pairs, p<0.001) as well as for Emboless-VC HD versus HDF (n=576 pairs, p=0.001). For the ‘Medium’ MBs the reduction in MBs for F5008-VC was better for HD versus HDF (n=576 pairs, p=0.013), but there was no difference for the Emboless-VC for HD versus HDF (n=319 pairs, p=0.239). For the ‘Large’ MBs the reduction in MBs for the F5008-VC was better for HD versus HDF (n=649 pairs, p=0.003) and for Emboless-VC better for HDF versus HD (n=613 pairs, p=0.009).

**Safety** – continued

User survey revealed only minor differences between mounting of the F5008-VC bloodline compared to the Emboless-VC bloodline. During mounting of the Emboless-VC bloodline, the need of adapter was an extra moment. During treatment, there were no differences regarding usability.

The side effects that the patients experienced during the dialysis sessions with the respective venous chambers did not differ between systems. Side effects included lower limb cramps, systolic blood pressure below 100 mm Hg and lower than at the start, and itching. Lower limb cramps appeared during the end of dialysis for 3 episodes (F5008-VC: n=2, Emboless-VC: n=1). The recovery time after dialysis was at a mean of 1.5 hours (median 0 hour in 62 of 74 reports, range 0-13), with no difference between the F5008-VC and the Emboless-VC.

Estimated clotting of the dialyzer after HD was similar between the venous chambers (Supplement Table 4). Clotting in the venous chamber was slight (Emboless-VC: one) or a visible clot (F5008-VC: one).

Supplement Table 4: Distribution of estimated clot grade in dialyzers after fulfilled dialysis procedure using either the standard Fresenius F5008-VC or Emboless-VC.

| Clot grade | Emboless-VC | F5008-VC | Total |
| --- | --- | --- | --- |
| None | 31 | 32 | 63 |
| Slight signs | 1 | 2 | 3 |
| Slight | 2 | 1 | 3 |
| Moderate | 0 | 1 | 1 |
| Data missing | 6 | 4 | 10 |
| Total | 40 | 40 | 80 |

The lowest systolic blood pressure did not differ between the F5008-VC (mean 128 ±23; median 121, 99-187 mmHg) and the Emboless-VC (mean 127 ±21; median 120, 94-185 mmHg). One dialysis was interrupted eight minutes before the planned end due to a lower blood pressure but without symptoms (F5008-VC: one). One patient had a systolic pressure at start of 99 lowered to 96 mmHg during HD without symptoms; few experienced any side effects due to the low pressure.

The pulse at the time of the lowest blood pressure did not differ between the F5008-VC (mean 71 ±14; median 70, 50-107) and the Emboless-VC (73 ±13; 74, 33-104 beats/min).

Besides air contamination of blood, laboratory estimates of change in leukocytes and platelets during HD is another biocompatibility measure. The study showed a similar and significant (p<0.01) reduction for both the F5008-VC and for the Emboless-VC at both 30 and 180 minutes for leukocytes, platelets, monocytes, lymphocytes, eosinophils, and neutrophils. Cell and platelets counts were adjusted for the effect of fluid infusion or removal by ultrafiltration for figures at 30min and 180min. There was no significant difference when using paired analysis between the Emboless-VC and the F5008-VC (Supplement Table 5).

Supplement Table 5: Median, IQR, and p-value of platelets and cells from start (_0min_) to 30min (_30min_) and 180min (_180min_), respectively, for the F5008-VC and the Emboless-VC. Samples were collected in 38 samples except for those marked (see foot note). Values achieved at 30min and 180min were corrected for volume changes by e.g., ultrafiltration, according to Schneditz et al. (2012). All values are given as x 10E9L^-1^ .

|  | **F5008-VC** | | | |  | **Emboless-VC** | | | |
| --- | --- | --- | --- | --- | --- | --- | --- | --- | --- |
| Variable |  | Percentiles | | P-value |  |  | Percentiles | | P-value |
|  | Median | 25th | 75th |  |  | Median | 25th | 75th |  |
| Platelets_0min_ | 194 | 162 | 246 | reference |  | 191 | 165 | 243 | reference |
| Platelets_30min_ | 173^1^ | 146 | 226 | <0.001 |  | 179 | 149 | 225 | <0.001 |
| Platelets_180min_ | 178^1^ | 146 | 224 | <0.001 |  | 169^2^ | 154 | 198 | <0.001 |
| Leukocytes_0min_ | 6.95 | 5.40 | 8.58 | reference |  | 6.45 | 5.58 | 8.53 | reference |
| Leukocytes_30min_ | 6.32^1^ | 5.15 | 7.57 | <0.001 |  | 5.84 | 4.95 | 7.38 | <0.001 |
| Leukocytes_180min_ | 6.21^1^ | 4.72 | 7.60 | <0.001 |  | 5.54^2^ | 4.33 | 6.77 | <0.001 |
| Neutrophils_0min_ | 4.05 | 3.48 | 5.70 | reference |  | 4.20 | 3.50 | 6.13 | reference |
| Neutrophils_30min_ | 4.10^1^ | 3.17 | 5.49 | 0.001 |  | 4.01 | 3.28 | 5.13 | 0.003 |
| Neutrophils_180min_ | 4.14^1^ | 2.99 | 5.18 | 0.046 |  | 3.41^2^ | 2.75 | 5.32 | 0.003 |
| Lymphocytes_0min_ | 1.20 | 0.70 | 1.70 | reference |  | 1.00 | 0.70 | 1.73 | reference |
| Lymphocytes_30min_ | 1.10^1^ | 0.75 | 1.70 | <0.001 |  | 1.05 | 0.59 | 1.59 | <0.001 |
| Lymphocytes_180min_ | 1.06^1^ | 0.48 | 1.58 | <0.001 |  | 1.06^2^ | 0.62 | 1.24 | 0.003 |
| Monocytes_0min_ | 0.60 | 0.50 | 0.80 | reference |  | 0.60 | 0.50 | 0.80 | reference |
| Monocytes_30min_ | 0.50^1^ | 0.39 | 0.61 | <0.001 |  | 0.47 | 0.40 | 0.55 | <0.001 |
| Monocytes_180min_ | 0.46^1^ | 0.37 | 0.59 | <0.001 |  | 0.41^2^ | 0.36 | 0.55 | <0.001 |
| Eosinophils_0min_ | 0.30 | 0.20 | 0.37 | reference |  | 0.20 | 0.20 | 0.30 | reference |
| Eosinophils_30min_ | 0.21^1^ | 0.20 | 0.29 | <0.001 |  | 0.20 | 0.17 | 0.24 | <0.001 |
| Eosinophils_180min_ | 0.19^1^ | 0.09 | 0.21 | <0.001 |  | 0.19^2^ | 0.10 | 0.21 | <0.001 |

^1^ N=37, ^2^ N=36 ;
